# P-2078. *Escherichia coli* Antimicrobial Resistance Risk Differences by Patient Demographics and Insurance Status in Rural Wisconsin

**DOI:** 10.1093/ofid/ofaf695.2242

**Published:** 2026-01-11

**Authors:** Laurel Legenza, Taylor A Wahlig, Maria Sundaram, Thomas R Fritsche, Sanjay Shukla, Joshua Petrie

**Affiliations:** University of Wisconsin-Madison School of Pharmacy, Madison, Wisconsin; Marshfield Clinic Health System, Marshfield, Wisconsin; Marshfield Clinic Research Institute, Marshfield, Wisconsin; Marshfield Clinic Health System, Marshfield, Wisconsin; Marshfield Clinic Research Institute, Marshfield, Wisconsin; Marshfield Clinic Research Institute, Marshfield, Wisconsin

## Abstract

**Background:**

Our prior research demonstrated *Escherichia coli* antimicrobial resistance differences at a neighborhood level from 2009 to 2018. As a first step in explaining this variation, we investigated patient factors relating to antimicrobial resistance.

**Figure.**
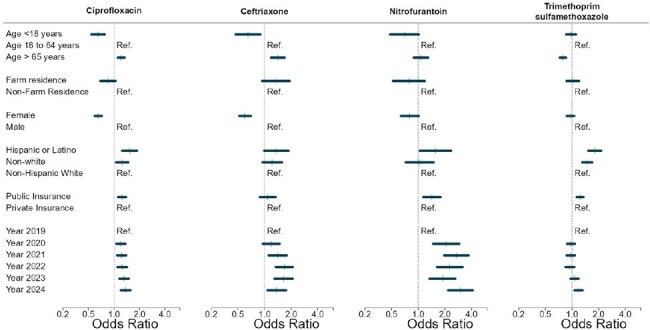
Patient Demographics and Insurance Status Predict E. coli Non-susceptibility to Antibiotics: Results from Multivariable Logistic Regression Models

**Methods:**

We conducted a retrospective analysis of patient demographics and *E. coli* antibiotic susceptibility from 2019 – 2024. Patients with a culture positive for *E. coli* and antibiotic susceptibility results at the Marshfield Clinic Health System were included. Patient-level variables analyzed included age, sex, race and ethnicity, type of insurance (public/private), farm residence (yes/no), and year of culture. We conducted logistic regression with generalized estimating equations analyses to identify predictors of *E. coli* susceptibility vs non-susceptibility (intermediate or resistant) to ciprofloxacin (CIP), nitrofurantoin (FM), trimethoprim/sulfamethoxazole (SXT), and ceftriaxone (CRO) as an indicator extended-spectrum beta-lactamase presence.

**Results:**

Regression results are presented in the Figure. Compared to adults 18 to 64 years, children were significantly less likely to have *E. coli* non-susceptible to CIP and CRO. Older adults (≥65 years) were less likely to have *E. coli* non- susceptible to SXT but more likely to have *E. coli* non-susceptible to CIP and CTX compared to younger adults. Females were less likely to have *E. coli* non-susceptible to CIP and CRO than males. Compared to white patients, odds of *E. coli* non-susceptibility were higher for Hispanic (CIP, SXT, and FM) and non-white patients (CIP and SXT). Public insurance was a predictor of *E. coli* non-susceptibility to CIP and FM. Odds of non-susceptibility were higher in 2024 compared to 2019 for all antibiotics. Of 36,043 unique *E. coli* isolates, 1,642 (4.6%) were from farm-residing patients; however, farm residence was not significantly associated with *E. coli* susceptibility in multivariable models.

**Conclusion:**

Children and female patients were at lower risk for non-susceptible *E. coli.* The risk of non-susceptibility was lower for SXT, and higher for CIP and CTX in older adults. Hispanic and non-white patients, and patients with public health insurance had greater risk for *E. coli* non-susceptibility.

**Disclosures:**

Maria Sundaram, PhD, MSPH, GSK: Grant/Research Support Joshua Petrie, PhD, CSL Seqirus: Advisor/Consultant|CSL Seqirus: Grant/Research Support|ModernaTX: Grant/Research Support

